# Glycine–phthalic acid (1/1)

**DOI:** 10.1107/S160053681204977X

**Published:** 2012-12-08

**Authors:** T. Balakrishnan, K. Ramamurthi, S. Thamotharan

**Affiliations:** a School of Physics, Bharathidasan University, Tiruchirappalli 620 024, India; b Department of Physics and Nanotechnology, SRM University, Kattankulathur 603 203, India; c Department of Bioinformatics, School of Chemical and Biotechnology, SASTRA University, Thanjavur 613 401, India

## Abstract

In the title compound, C_2_H_5_NO_2_·C_8_H_6_O_4_, the glycine mol­ecule exists as a zwitterion (2-aza­niumyl­ethano­ate) with a positively charged amino group and a negatively charged carboxyl­ate group. In the crystal, N—H⋯O and O—H⋯O hydrogen bonds link the components into layers parallel to the *ab* plane. The central part of each layer is composed of hydrogen-bonded glycine zwitterions, while phthalic acid mol­ecules inter­act with the zwitterions in such a way that benzene rings protrude up and down from the layer.

## Related literature

For related structures, see: Losev *et al.* (2011 ▶); Herbstein *et al.* (1981 ▶). For graph-set motifs, see: Bernstein *et al.* (1995 ▶). For head-to-tail hydrogen bonds, see: Sharma *et al.* (2006 ▶); Selvaraj *et al.* (2007 ▶).
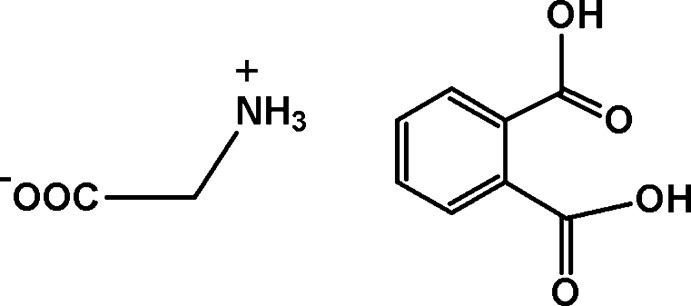


## Experimental

### 

#### Crystal data

C_2_H_5_NO_2_·C_8_H_6_O_4_
*M* *_r_* = 241.20
Orthorhombic, 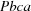

*a* = 7.9657 (5) Å
*b* = 11.3470 (7) Å
*c* = 23.513 (2) Å
*V* = 2125.3 (3) Å^3^
*Z* = 8
Mo *K*α radiation
μ = 0.13 mm^−1^
*T* = 173 K
0.53 × 0.46 × 0.30 mm

#### Data collection

Stoe IPDS diffractometer
15716 measured reflections
2077 independent reflections
1597 reflections with *I* > 2σ(*I*)
*R*_int_ = 0.041

#### Refinement

*R*[*F* ^2^ > 2σ(*F* ^2^)] = 0.034
*wR*(*F* ^2^) = 0.090
*S* = 1.01
2077 reflections
175 parameters
H atoms treated by a mixture of independent and constrained refinement
Δρ_max_ = 0.22 e Å^−3^
Δρ_min_ = −0.19 e Å^−3^

### 

Data collection: *EXPOSE* in *IPDS-I Software* (Stoe & Cie, 2000 ▶); cell refinement: *CELL* in *IPDS-I Software*; data reduction: *INTEGRATE* in *IPDS-I Software*; program(s) used to solve structure: *SHELXS97* (Sheldrick, 2008 ▶); program(s) used to refine structure: *SHELXL97* (Sheldrick, 2008 ▶); molecular graphics: *PLATON* (Spek, 2009 ▶) and *ORTEP-3 for Windows* (Farrugia, 2012 ▶); software used to prepare material for publication: *SHELXL97*.

## Supplementary Material

Crystal structure: contains datablock(s) I, global. DOI: 10.1107/S160053681204977X/cv5360sup1.cif

Structure factors: contains datablock(s) I. DOI: 10.1107/S160053681204977X/cv5360Isup2.hkl

Supplementary material file. DOI: 10.1107/S160053681204977X/cv5360Isup3.cml

Additional supplementary materials:  crystallographic information; 3D view; checkCIF report

## Figures and Tables

**Table 1:** Hydrogen-bond geometry (Å, °)

*D*—H⋯*A*	*D*—H	H⋯*A*	*D*⋯*A*	*D*—H⋯*A*
N1—H1*A*⋯O5^i^	0.917 (19)	1.992 (19)	2.8398 (16)	153.0 (16)
N1—H1*B*⋯O3^ii^	0.91 (2)	2.13 (2)	3.0219 (16)	164.6 (17)
N1—H1*C*⋯O2^iii^	0.88 (2)	2.181 (19)	2.8934 (16)	137.4 (15)
N1—H1*C*⋯O3^iv^	0.88 (2)	2.416 (19)	3.0681 (16)	130.9 (15)
O4—H4*O*⋯O2^i^	0.96 (3)	1.58 (3)	2.5383 (14)	175 (2)
O6—H6*O*⋯O1^v^	0.98 (2)	1.56 (2)	2.5337 (13)	171 (2)

Symmetry codes: (i) 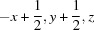; (ii) 
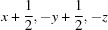
; (iii) 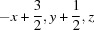; (iv) 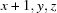; (v) 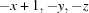.

**Fig. 1.**
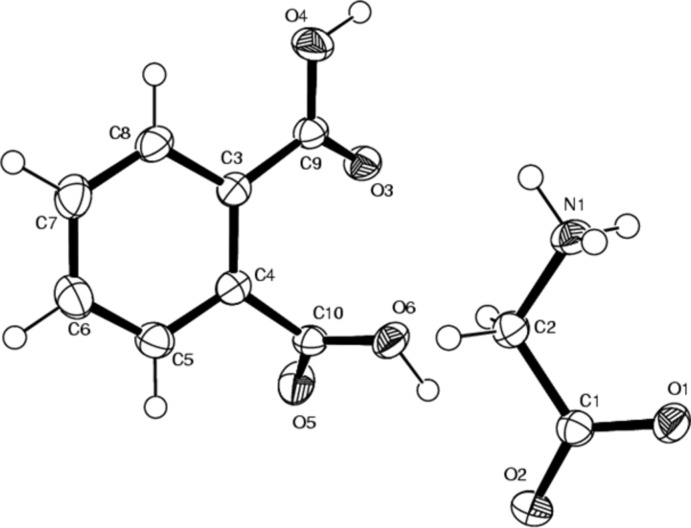
A content of asymmetric unit of (I) showing the atomic-numbering scheme. Displacement ellipsoids are drawn at the 50% probability level.

**Fig. 2.**
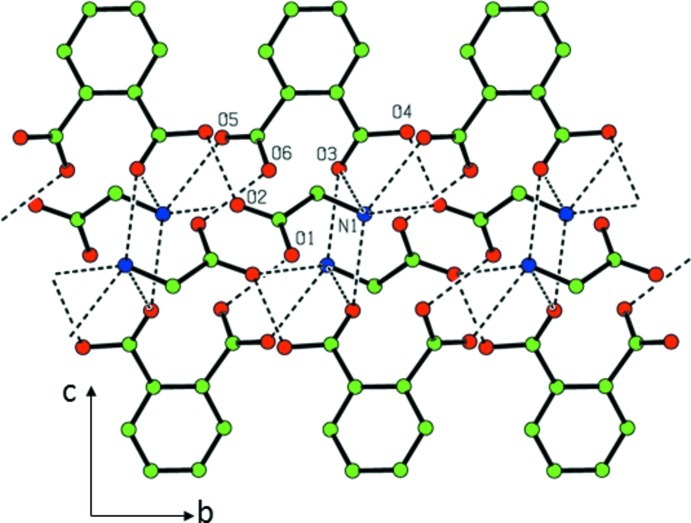
Basic aggregation pattern in (I) viewed in [100]. Dashed lines denote hydrogen bonds. H atoms have been omitted for clarity.

**Fig. 3.**
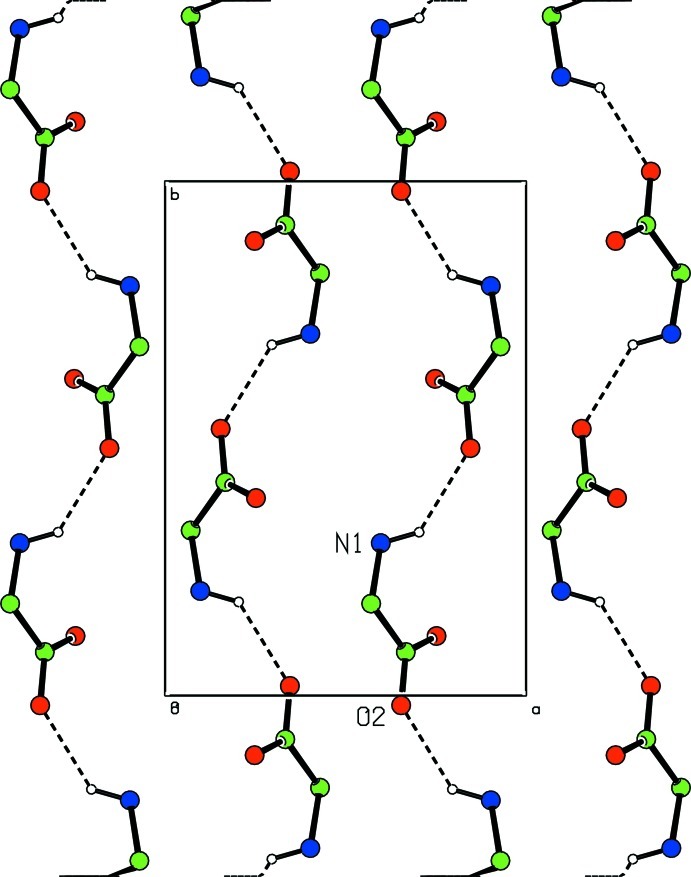
Head-to-tail sequences of zwitterionic glycine molecules in (I) viewed in [001]. Dashed lines denote hydrogen bonds. H atoms not involved in H-bonding were omitted for clarity.

## supplementary crystallographic information

### Comment

As part of our studies on amino acids and carboxylic acids interactions (Sharma *et al.* 2006; Selvaraj *et al.*, 2007), we report here the crystal structure of the title cocrystal of glycine and phthalic acid, (I).

The asymmetric unit of (I) contains one glycine molecule and one phthalic acid molecule (Fig. 1). The glycine molecule exists as a zwitterion with a positively charged amino group and a negatively charged carboxylate group as found in glycine-trimesic acid complex (Herbstein *et al.*, 1981) and glycine-glutaric acid cocrystal (Losev *et al.*, 2011), where glutaric acid exists as a neutral molecule. The phthalic acid exists as a neutral molecule with both carboxylic acid groups being unionized. The stoichiometry between the glycine and phthalic acid is 1:1.

The crystal packing is stabilized by a network of N—H···O and O—H···O hydrogen bonds (Table 1). As illustrated in Fig. 2, the basic aggregation pattern observed in the complex is a layered architecture of zwitterionic glycine and neutral phthalic acid molecules. An antiparallel linear array of zwitterionic glycines are sandwiched between phthalic acid layers.

In (I), the zwitterionic glycine has one donor atom capable of forming three hydrogen bonds, and one of them forms bifurcated hydrogen bonds, while neutral phthalic acid can also forms three hydrogen bonds through two acceptors (Table 1). In the crystal structure, the zwitterionic glycines are arranged in linear arrays along [010] direction. In each array, adjacent glycines are connected by a N1···O2 hydrogen bond which can be described as a head-to-tail sequence having a graph-set motif of C5 (Bernstein *et al.*, 1995) (Fig. 3). In contrast to (I), no head-to-tail sequence was observed in glycine-glutaric acid cocrystal (Losev *et al.*, 2011). As observed in many binary complexes of amino acids complexed with carboxylic acids, the neutral molecules in the complex do not interact among themselves. However, here, phthalic acid molecule is interconnected by zwitterionic glycines *via* two intermolecular N1···O3 hydrogen bonds. The glycine amino group acts as donor for 1-substituted carboxylic O3 atoms of the phthalic acid molecules emanating from different phthalic acids layers. Another carboxylic O5 atom acts as acceptor for an intermolecular hydrogen bond with the amino group of a glycine. The 2-substituted carboxylic group of the phthalic acid molecules in two different layers are interconnected by glycines. One carboxylic group in one layer interacts with the glycine in one layer, while its symmetry-related equivalents in the adjacent layers interacts with the glycine in the neighbouring layer [C^1^_2_(4) graph-set motif]. The donor atoms (O4 and O6) of the phthalic acid molecule participate in intermolecular short and linear O—H···O hydrogen bonds with the carboxylate group of glycine. These hydrogen bonds produce C^2^_2_(11) chains that run parallel to the *a* axis.

### Experimental

The title complex was prepared by dissolving glycine and phthalic acid in a stoichiometric ratio in double distilled water. The resulting solution was heated to *ca* 50° C and the title cocrystal was obtained by a slow cooling method from an aqueous solution.

### Refinement

The H-atoms bound to nitrogen and oxygen were located from difference electron density maps and isotropically refined. All the remaining H atoms were placed in geometrically idealized positions (C—H = 0.95-0.99 Å) and constrained to ride on their parent atoms, with U_iso_(H) = 1.2U_eq_(C).

### Crystal data

**Table:**

C_2_H_5_NO_2_·C_8_H_6_O_4_	*F*(000) = 1008
*M**_r_* = 241.20	*D*_x_ = 1.508 Mg m^−^^3^
Orthorhombic, *P**b**c**a*	Mo *K*α radiation, λ = 0.71073 Å
Hall symbol: -P 2ac 2ab	Cell parameters from 8000 reflections
*a* = 7.9657 (5) Å	θ = 2.6–26.1°
*b* = 11.3470 (7) Å	µ = 0.13 mm^−^^1^
*c* = 23.513 (2) Å	*T* = 173 K
*V* = 2125.3 (3) Å^3^	Block, colourless
*Z* = 8	0.53 × 0.46 × 0.30 mm

### Data collection

**Table:**

Stoe IPDS diffractometer	1597 reflections with *I* > 2σ(*I*)
Radiation source: fine-focus sealed tube	*R*_int_ = 0.041
Graphite monochromator	θ_max_ = 26.0°, θ_min_ = 3.1°
Detector resolution: 0.81Å pixels mm^-1^	*h* = −9→9
phi rotation scans	*k* = −13→13
15716 measured reflections	*l* = −29→28
2077 independent reflections	

### Refinement

**Table:**

Refinement on *F*^2^	Secondary atom site location: difference Fourier map
Least-squares matrix: full	Hydrogen site location: inferred from neighbouring sites
*R*[*F*^2^ > 2σ(*F*^2^)] = 0.034	H atoms treated by a mixture of independent and constrained refinement
*wR*(*F*^2^) = 0.090	*w* = 1/[σ^2^(*F*_o_^2^) + (0.064*P*)^2^] where *P* = (*F*_o_^2^ + 2*F*_c_^2^)/3
*S* = 1.01	(Δ/σ)_max_ < 0.001
2077 reflections	Δρ_max_ = 0.22 e Å^−^^3^
175 parameters	Δρ_min_ = −0.19 e Å^−^^3^
0 restraints	Extinction correction: *SHELXL97* (Sheldrick, 2008), Fc^*^=kFc[1+0.001xFc^2^λ^3^/sin(2θ)]^-1/4^
Primary atom site location: structure-invariant direct methods	Extinction coefficient: 0.0086 (15)

### Special details

**Table:**

Geometry. All esds (except the esd in the dihedral angle between two l.s. planes) are estimated using the full covariance matrix. The cell esds are taken into account individually in the estimation of esds in distances, angles and torsion angles; correlations between esds in cell parameters are only used when they are defined by crystal symmetry. An approximate (isotropic) treatment of cell esds is used for estimating esds involving l.s. planes.
Refinement. Refinement of F^2^ against ALL reflections. The weighted R-factor wR and goodness of fit S are based on F^2^, conventional R-factors R are based on F, with F set to zero for negative F^2^. The threshold expression of F^2^ > 2sigma(F^2^) is used only for calculating R-factors(gt) etc. and is not relevant to the choice of reflections for refinement. R-factors based on F^2^ are statistically about twice as large as those based on F, and R- factors based on ALL data will be even larger.

### Fractional atomic coordinates and isotropic or equivalent isotropic displacement parameters (Å^2^)

**Table:**

	*x*	*y*	*z*	*U*_iso_*/*U*_eq_	
O3	−0.06389 (12)	0.23293 (9)	0.08480 (4)	0.0293 (3)	
O4	−0.00401 (15)	0.40181 (9)	0.12875 (4)	0.0374 (3)	
H4O	−0.058 (3)	0.435 (2)	0.0959 (11)	0.075 (7)*	
O5	0.00354 (13)	−0.05643 (9)	0.12213 (4)	0.0365 (3)	
O6	0.19779 (12)	0.05907 (9)	0.08314 (4)	0.0311 (3)	
H6O	0.205 (3)	−0.008 (2)	0.0572 (10)	0.072 (6)*	
C3	0.06342 (15)	0.22668 (12)	0.17621 (5)	0.0238 (3)	
C4	0.10175 (15)	0.10660 (12)	0.17542 (5)	0.0242 (3)	
C5	0.15369 (17)	0.05214 (13)	0.22514 (6)	0.0294 (3)	
H5	0.1795	−0.0296	0.2247	0.035*	
C6	0.16855 (19)	0.11483 (14)	0.27541 (6)	0.0347 (3)	
H6	0.2054	0.0764	0.3091	0.042*	
C7	0.12967 (18)	0.23317 (14)	0.27639 (6)	0.0336 (3)	
H7	0.1387	0.2765	0.3108	0.040*	
C8	0.07751 (17)	0.28854 (13)	0.22713 (6)	0.0278 (3)	
H8	0.0508	0.3701	0.2280	0.033*	
C9	−0.00603 (16)	0.28671 (12)	0.12521 (5)	0.0249 (3)	
C10	0.09312 (16)	0.03014 (11)	0.12370 (5)	0.0247 (3)	
O1	0.75187 (11)	0.11587 (8)	−0.01838 (4)	0.0269 (2)	
O2	0.65409 (12)	−0.01881 (8)	0.04157 (4)	0.0317 (3)	
N1	0.59761 (16)	0.29647 (10)	0.03063 (5)	0.0258 (3)	
H1A	0.543 (2)	0.3538 (16)	0.0511 (8)	0.042 (5)*	
H1B	0.560 (2)	0.2998 (17)	−0.0061 (9)	0.051 (5)*	
H1C	0.704 (3)	0.3178 (16)	0.0329 (8)	0.043 (5)*	
C1	0.66689 (15)	0.08474 (11)	0.02398 (5)	0.0226 (3)	
C2	0.57097 (16)	0.17890 (11)	0.05551 (6)	0.0249 (3)	
H2A	0.4497	0.1599	0.0546	0.030*	
H2B	0.6072	0.1798	0.0958	0.030*	

### Atomic displacement parameters (Å^2^)

**Table:**

	*U*^11^	*U*^22^	*U*^33^	*U*^12^	*U*^13^	*U*^23^
O3	0.0348 (5)	0.0245 (5)	0.0286 (5)	−0.0013 (4)	−0.0052 (4)	−0.0042 (4)
O4	0.0615 (7)	0.0201 (6)	0.0307 (6)	−0.0003 (5)	−0.0090 (5)	−0.0012 (4)
O5	0.0476 (6)	0.0296 (6)	0.0322 (6)	−0.0152 (5)	0.0070 (4)	−0.0067 (4)
O6	0.0368 (5)	0.0226 (5)	0.0338 (5)	−0.0036 (4)	0.0124 (4)	−0.0055 (4)
C3	0.0231 (6)	0.0230 (7)	0.0251 (7)	−0.0023 (5)	0.0022 (5)	−0.0020 (5)
C4	0.0215 (6)	0.0229 (7)	0.0280 (7)	−0.0032 (5)	0.0035 (5)	−0.0018 (5)
C5	0.0318 (7)	0.0250 (7)	0.0315 (7)	0.0004 (6)	0.0006 (5)	0.0024 (6)
C6	0.0398 (8)	0.0363 (9)	0.0279 (7)	−0.0013 (6)	−0.0036 (6)	0.0047 (6)
C7	0.0408 (8)	0.0355 (9)	0.0245 (7)	−0.0047 (7)	−0.0017 (6)	−0.0044 (6)
C8	0.0313 (7)	0.0239 (7)	0.0283 (7)	−0.0016 (5)	0.0009 (5)	−0.0055 (5)
C9	0.0269 (6)	0.0213 (7)	0.0266 (7)	−0.0011 (5)	0.0014 (5)	−0.0026 (5)
C10	0.0278 (6)	0.0186 (7)	0.0277 (7)	−0.0001 (5)	0.0021 (5)	−0.0004 (5)
O1	0.0323 (5)	0.0227 (5)	0.0256 (5)	−0.0013 (4)	0.0069 (4)	−0.0038 (4)
O2	0.0415 (6)	0.0204 (5)	0.0332 (5)	0.0037 (4)	0.0057 (4)	0.0041 (4)
N1	0.0293 (6)	0.0192 (6)	0.0288 (6)	0.0010 (5)	0.0024 (5)	−0.0043 (5)
C1	0.0244 (6)	0.0211 (7)	0.0223 (6)	−0.0002 (5)	−0.0017 (5)	−0.0016 (5)
C2	0.0283 (7)	0.0216 (7)	0.0247 (6)	−0.0002 (5)	0.0040 (5)	−0.0012 (5)

### Geometric parameters (Å, º)

**Table:**

O3—C9	1.2197 (15)	C6—H6	0.9500
O4—C9	1.3088 (18)	C7—C8	1.382 (2)
O4—H4O	0.96 (3)	C7—H7	0.9500
O5—C10	1.2147 (16)	C8—H8	0.9500
O6—C10	1.3086 (16)	O1—C1	1.2550 (15)
O6—H6O	0.98 (2)	O2—C1	1.2498 (16)
C3—C8	1.3925 (18)	N1—C2	1.4720 (17)
C3—C4	1.396 (2)	N1—H1A	0.917 (19)
C3—C9	1.4859 (18)	N1—H1B	0.91 (2)
C4—C5	1.3855 (19)	N1—H1C	0.88 (2)
C4—C10	1.4955 (18)	C1—C2	1.5083 (18)
C5—C6	1.385 (2)	C2—H2A	0.9900
C5—H5	0.9500	C2—H2B	0.9900
C6—C7	1.378 (2)		
			
C9—O4—H4O	109.8 (14)	O3—C9—C3	122.68 (13)
C10—O6—H6O	107.5 (13)	O4—C9—C3	113.67 (11)
C8—C3—C4	119.06 (12)	O5—C10—O6	123.72 (12)
C8—C3—C9	119.52 (12)	O5—C10—C4	121.39 (11)
C4—C3—C9	121.20 (11)	O6—C10—C4	114.70 (11)
C5—C4—C3	119.29 (12)	C2—N1—H1A	111.5 (11)
C5—C4—C10	116.18 (12)	C2—N1—H1B	111.5 (12)
C3—C4—C10	124.53 (12)	H1A—N1—H1B	108.2 (16)
C6—C5—C4	121.11 (13)	C2—N1—H1C	111.3 (12)
C6—C5—H5	119.4	H1A—N1—H1C	103.1 (16)
C4—C5—H5	119.4	H1B—N1—H1C	110.9 (17)
C7—C6—C5	119.71 (13)	O2—C1—O1	124.83 (12)
C7—C6—H6	120.1	O2—C1—C2	117.52 (11)
C5—C6—H6	120.1	O1—C1—C2	117.64 (11)
C6—C7—C8	119.78 (13)	N1—C2—C1	111.94 (11)
C6—C7—H7	120.1	N1—C2—H2A	109.2
C8—C7—H7	120.1	C1—C2—H2A	109.2
C7—C8—C3	121.05 (13)	N1—C2—H2B	109.2
C7—C8—H8	119.5	C1—C2—H2B	109.2
C3—C8—H8	119.5	H2A—C2—H2B	107.9
O3—C9—O4	123.61 (13)		
			
C8—C3—C4—C5	−0.36 (18)	C8—C3—C9—O3	−159.60 (13)
C9—C3—C4—C5	−174.93 (12)	C4—C3—C9—O3	14.95 (19)
C8—C3—C4—C10	−179.55 (12)	C8—C3—C9—O4	18.29 (17)
C9—C3—C4—C10	5.88 (19)	C4—C3—C9—O4	−167.16 (12)
C3—C4—C5—C6	−0.2 (2)	C5—C4—C10—O5	59.45 (18)
C10—C4—C5—C6	179.09 (12)	C3—C4—C10—O5	−121.34 (15)
C4—C5—C6—C7	0.6 (2)	C5—C4—C10—O6	−115.59 (13)
C5—C6—C7—C8	−0.5 (2)	C3—C4—C10—O6	63.62 (17)
C6—C7—C8—C3	0.0 (2)	O2—C1—C2—N1	179.74 (11)
C4—C3—C8—C7	0.46 (19)	O1—C1—C2—N1	−1.15 (17)
C9—C3—C8—C7	175.12 (12)		

### Hydrogen-bond geometry (Å, º)

**Table:**

*D*—H···*A*	*D*—H	H···*A*	*D*···*A*	*D*—H···*A*
N1—H1*A*···O5^i^	0.917 (19)	1.992 (19)	2.8398 (16)	153.0 (16)
N1—H1*B*···O3^ii^	0.91 (2)	2.13 (2)	3.0219 (16)	164.6 (17)
N1—H1*C*···O2^iii^	0.88 (2)	2.181 (19)	2.8934 (16)	137.4 (15)
N1—H1*C*···O3^iv^	0.88 (2)	2.416 (19)	3.0681 (16)	130.9 (15)
O4—H4*O*···O2^i^	0.96 (3)	1.58 (3)	2.5383 (14)	175 (2)
O6—H6*O*···O1^v^	0.98 (2)	1.56 (2)	2.5337 (13)	171 (2)

Symmetry codes: (i) −*x*+1/2, *y*+1/2, *z*; (ii) *x*+1/2, −*y*+1/2, −*z*; (iii) −*x*+3/2, *y*+1/2, *z*; (iv) *x*+1, *y*, *z*; (v) −*x*+1, −*y*, −*z*.
